# Dental Treatment Needs in Older Adults Undergoing Acute Geriatric Care: A Cross-Sectional Observational Study of Clinical Need and Patient-Reported Wish for Dental Treatment

**DOI:** 10.3390/jcm15103610

**Published:** 2026-05-08

**Authors:** Laura Isabell Werneburg, Aline Schönenberg, Stefanie Andernach, Jeremias Hey, Monika Kasaliyska, Tino Prell, Ramona Schweyen

**Affiliations:** 1 Department of Prosthodontics and Geriatric Dentistry, Martin Luther University Halle-Wittenberg, 06112 Halle (Saale), Germany; laura.werneburg@uk-halle.de (L.I.W.); jeremias.hey@uk-halle.de (J.H.); monika.kasaliyska@uk-halle.de (M.K.); 2 Department of Geriatrics, Jena University Hospital, 07747 Jena, Germany; aline.schoenenberg@med.uni-jena.de (A.S.); tino.prell@med.uni-jena.de (T.P.); 3 Department of Geriatrics, Martin Luther University Halle-Wittenberg, 06120 Halle (Saale), Germany; stefanie.andernach@uk-halle.de

**Keywords:** geriatric dentistry, aged, inpatients, dental care, treatment need, patient preference, oral health, quality of life, GOHAI

## Abstract

**Background/Objectives**: Dental care is not routinely integrated into acute geriatric complex treatment, although older inpatients often present with substantial oral disease. This study assessed dental treatment needs, their relationship with patients’ expressed wish for dental treatment, and their association with oral health-related quality of life (OHRQoL). **Methods**: In this cross-sectional observational study, patients undergoing acute geriatric complex treatment at a university hospital in Germany (April 2023 to September 2024) were included if able to provide valid self-reported data. Standardized bedside dental examinations were performed by calibrated dentists without radiographs. Treatment needs were categorized as restorative, periodontal, surgical, and prosthodontic. General health and geriatric data were obtained from medical records. Patients reported their wish for dental treatment and completed the General Oral Health Assessment Index (GOHAI). **Results**: A total of 214 hospitalized older adults (mean age 82 ± 6 years; 58.4% female) were included. Overall, 94.9% showed clinically assessed dental treatment needs; however, most were elective or functionally relevant, while medically urgent needs (e.g., extraction indications) were identified in 26.7% of dentate patients. The most frequent categories were periodontal (93.2%) and prosthodontic (67.7%) treatment need. No significant association was found between clinically assessed treatment need and patient-reported wish for dental treatment. In exploratory analyses, surgical treatment need and supportive living environments were associated with treatment wish, although model fit was limited. Prosthodontic treatment need, lower functional oral intake, and institutional living were associated with lower OHRQoL. **Conclusions**: Dental treatment needs are highly prevalent in older adults undergoing acute geriatric complex treatment but are not consistently reflected by patient-reported wish for treatment during hospitalization. These findings indicate a mismatch between clinical assessment and patient-reported preferences. Interpretation is limited by the cross-sectional design and the use of a single dichotomous measure of treatment wish.

## 1. Introduction

Due to demographic change, the proportion of older adults in the population is increasing, requiring adaptations in medical and dental healthcare systems [1,2]. In Germany, individuals are entitled to needs-based healthcare irrespective of age, disability, frailty, or care dependency [3]. However, the heterogeneity of older adults poses challenges for providing timely and appropriate care. These challenges are further compounded by rapid transitions between health states (e.g., from fit to frail or care-dependent), which require flexible and interdisciplinary care approaches [4].

From a dental perspective, adequate care for older adults in advanced stages of life is often challenging, particularly in acute hospital and geriatric care settings where dental services are not routinely integrated [5,6]. This is further complicated by barriers to accessing routine dental care, especially among older adults with multimorbidity [7]. Consequently, unmet dental needs are common in frail and care-dependent populations.

Epidemiological data suggest that documented dental treatment need decreases with increasing age, which may reflect underutilization of dental services rather than improved oral health [8]. Accordingly, oral health is often poorer among frail and care-dependent older adults compared with their fitter counterparts [9].

Poor oral health has been associated with systemic conditions such as cardiovascular disease, pneumonia, and diabetes [10,11,12,13], which are highly relevant in hospitalized older patients. In addition, higher age and declining oral health are linked to reduced oral health-related quality of life (OHRQoL) [14]. OHRQoL can be assessed using validated self-report instruments; in geriatric populations, the General Oral Health Assessment Index (GOHAI) is widely used and internationally established [15].

The German healthcare system faces the challenge of systematically identifying vulnerable older adults, particularly those with care dependency, and facilitating their access to appropriate dental care [8]. In this context, acute geriatric complex treatment has emerged as an established model of care for older, often multimorbid patients requiring inpatient treatment following acute illness [16]. This setting is particularly relevant, as patients are at high risk of functional decline and are already managed by an interdisciplinary team aimed at preserving mobility, independence, and quality of life.

Despite this comprehensive care approach, dental assessment and treatment are not routinely integrated into acute geriatric complex treatment [8,17]. This represents a critical gap, as hospitalization may provide a unique opportunity to identify and address previously unmet dental needs in a vulnerable population.

However, it remains unclear to what extent clinically relevant dental treatment needs are present in this setting and whether these needs correspond to patients’ expressed wish for dental treatment during acute hospitalization. Clinically assessed dental treatment need represents a normative evaluation based on professional criteria, independent of patient perception. In this context, different types of need can be distinguished, including urgent or medically relevant needs (e.g., extraction of severely destroyed teeth as potential sources of infection), functionally relevant but non-urgent needs (e.g., insufficient prostheses affecting oral intake), and elective or preventive needs (e.g., professional tooth cleaning).

In contrast, patient-reported measures may reflect perceived need, expressed desire, or actual demand, which are influenced by individual priorities, health status, and situational constraints during acute illness. These constructs are not interchangeable and may diverge substantially, particularly in frail and hospitalized populations. From a clinical perspective, it is generally important to assess whether patients consider dental treatment desirable during acute geriatric care. Accordingly, the present study focuses on the relationship between clinically assessed treatment need and patient-reported wish for dental treatment as captured during acute hospitalization, without assuming equivalence between the above mentioned dimensions.

The aim of this cross-sectional clinical study was to assess the prevalence of clinically determined dental treatment needs in older adults undergoing acute geriatric complex treatment, to examine their relationship with patient-reported wish for dental treatment, and to analyze their association with OHRQoL.

## 2. Materials and Methods

### 2.1. Study Design

This observational clinical study was conducted between April 2023 and September 2024 at the Department of Geriatrics, Halle University Hospital, Halle (Saale), Germany. Participation was voluntary.

Patients were invited to participate if they were undergoing early complex geriatric rehabilitation treatment for older adults hospitalized with acute illness or injury, classified under the German Operations and Procedures Key (OPS) code 8–550.1 [17,18]. Eligibility criteria for this treatment typically included an age of ≥70 years in combination with geriatric multimorbidity, or an age of ≥60 years with pronounced multimorbidity. For patients aged 60–69 years, a higher degree of multimorbidity was required, whereas in patients aged ≥80 years, multimorbidity was not mandatory due to assumed increased vulnerability. Indicators of such vulnerability included pre-existing care dependency, complications during hospitalization, cognitive impairment, or an increased need for assistance with activities of daily living. Early complex geriatric rehabilitation treatment typically spans 14 to 21 days and is delivered by a multidisciplinary team including geriatricians, nurses, physiotherapists, occupational therapists, speech and language therapists, social workers, psychologists, and other specialists [16,17]. Dental examinations reported in the present study were routinely performed during the second week of the inpatient stay.

Patients were excluded if they were unable to provide valid self-reported data due to severe cognitive impairment, such as delirium or advanced dementia. This exclusion was necessary because key study outcomes, including wish for dental treatment and oral health-related quality of life (OHRQoL), relied on patient-reported measures. If patients’ cognitive status improved during the course of treatment, they were offered the opportunity to participate toward the end of their hospital stay.

A total of 435 patients admitted for acute geriatric complex treatment were screened. In 115 cases, participation was not pursued due to time constraints, including high therapeutic workload, early discharge, or short duration since admission. Of the remaining 320 potentially eligible patients, 104 were excluded, including 35 due to severe cognitive impairment and 69 due to other severe health problems. Consequently, 216 patients were enrolled in the study. In 2 cases, assessments could not be completed, resulting in a final analyzed sample of 214 patients.

All participants provided written informed consent. The study was approved by the local ethics committee of the University Hospital Halle (number 2022-026; initial approval on 24 May 2022, with amendments on 28 May 2024, and 4 July 2025).

### 2.2. Variables of Interest

The following variables of interest and covariates were selected:

#### 2.2.1. Dental Status and Clinically Assessed Dental Treatment Need

In addition to the clinical findings and routine geriatric assessments obtained during early complex rehabilitation treatment, all patients underwent a standardized dental examination in their hospital rooms using a headlamp. No dental radiographs were obtained as part of the study protocol. Examinations were conducted by two experienced, calibrated dentists affiliated with the University Clinic for Prosthodontics and Geriatric Dentistry. The assessment was performed using a basic dental examination set (mouth mirror, explorer/probe, and tweezers), complemented by a periodontal probe and cold spray for sensibility testing; where clinically indicated, articulating paper, indicator silicone, and a spatula were additionally employed. Prior to study initiation, both examiners were calibrated through the independent assessment of the same group of ten patients. The findings were subsequently compared and discussed to ensure consistency in diagnostic criteria and to improve inter-examiner reliability.

Documentation included the presence, absence, and replacement of teeth; carious lesions; the Periodontal Screening Index (PSI) [19], a standardized screening tool assessing periodontal status per sextant based on bleeding on probing, calculus, and probing depth, with scores ranging from 0 (healthy periodontal conditions) to 4 (deep periodontal pockets ≥6 mm), where the highest score per sextant is recorded; and the Approximal Plaque Index (API) [20], which quantifies oral hygiene by recording the percentage of plaque-positive approximal sites, with values ≤25% indicating good oral hygiene and values above approximately 35% indicating insufficient oral hygiene. In the presence of fixed dental prostheses (FPDs), crown margins were assessed for adequacy using a dental probe, and any defects of the veneering material were recorded. For removable dental prostheses (RPDs), the fit of the denture base, the condition of the artificial teeth, the presence of fractures or chipping, and the adequacy of retention elements were evaluated.

Afterwards, dental treatment needs were classified as follows:

#### 2.2.2. Elective or Preventive Needs

- Periodontal treatment need: classified as “no need”, “professional tooth cleaning (PTC) indicated” (PSI score of ≥2 and/or API > 35%), or “systematic periodontal therapy (SPT) indicated” (PSI score of ≥3).
- Restorative treatment need: classified as “no need” or “restorative treatment indicated”, defined by the presence of probe-detectable carious lesions in teeth without partial or full crowns.

#### 2.2.3. Functionally Relevant but Non-Urgent Needs

- Prosthodontic treatment need: classified as “no need”, “repair/relining indicated” (e.g., relining, replacement of artificial teeth or attachment components in case of removable partial dentures [RPD]), “new fixed partial dentures [FPD] indicated” [e.g., in cases of advanced marginal caries], “new RPD indicated” [in cases of non-functional prostheses not amenable to technical repair], “combined FPD and RPD treatment indicated”, or “not assessable” (in patients whose prostheses were not available in the hospital during the inpatient stay).

#### 2.2.4. Urgent or Medically Relevant Needs

- Surgical treatment need: classified as “no need” or “extraction indicated”, defined by teeth with advanced periodontal disease (PSI score of 4) and marked mobility, advanced destruction of the clinical crown due to caries, or the presence of retained roots in combination with subjective complaints.

Based on these findings, the DMFT index (Decayed, Missing, Filled Teeth) was calculated for each patient. In addition, dentition status was classified as “fully dentate” (all teeth present, excluding third molars), “partially dentate” (at least one natural tooth present but fewer than 28 teeth), or “edentulous” (no remaining natural teeth or only residual roots covered by mucosa or prosthetic structures). Overall dental treatment need was finally classified as “no” (no periodontal, restorative, surgical, or prosthodontic treatment need identified) or “yes” (at least one subtype of dental treatment need identified).

#### 2.2.5. Patient-Reported Desire for Dental Treatment and OHRQoL

Patients were asked about their current desire for dental treatment (“yes, I would like treatment in the near future” vs. “no, I do not want treatment”). In addition, patients were asked to complete the General Oral Health Assessment Index (GOHAI) questionnaire [21]. The German version of the GOHAI has been previously validated and demonstrated good reliability and validity in geriatric populations. The 12 GOHAI items capture three domains: physical function (eating, speaking, and swallowing; items 1–4), psychosocial functioning (self-esteem, social withdrawal, and concerns regarding oral health; items 6, 7, 9–11), and symptoms associated with oral disease (use of analgesic medication and oral discomfort; items 5, 8, and 12). All items were answered on a five-point Likert scale (1 = “very often”, 2 = “often”, 3 = “sometimes”, 4 = “seldom”, 5 = “never”). Responses to positively worded items (3, 5, and 7) were reverse-coded, and item scores were summed to obtain the total GOHAI score (range: 12–60). Higher GOHAI score indicates better oral health–related quality of life (OHRQoL). For the GOHAI, the minimal important difference (MID)—defined as the smallest score difference considered clinically meaningful with respect to OHRQoL—was set at 3 points [22]. This threshold was used descriptively to support the interpretation of clinical relevance and was not applied as a criterion in statistical analyses. In cases where patients were unable to complete the questionnaire independently due to visual or motor impairments, assistance was provided where necessary to ensure valid completion.

### 2.3. Independent Variables (Covariates)

The selection of covariates was based on their established relevance in geriatric assessment and their potential influence on patient-reported wish for dental treatment as well as OHRQoL. Variables were chosen to capture key domains including demographic characteristics, multimorbidity, functional status, frailty, psychological status, living situation, and nutritional/oral intake status:

#### 2.3.1. General Health Characteristics

- Chronological age (years) and sex (male/female);
- Level of care prior to hospital admission as an indicator of long-term care needs and assistance requirements (range: 1 = minor impairment of independence or functional abilities to 5 = most severe impairment with special requirements for nursing care);
- Pre-admission living arrangement (living alone; living alone with support from friends or family; living with a partner or family; receiving outpatient/home nursing care; residing in institutional long-term care);
- Number of different medications taken daily.

#### 2.3.2. Geriatric and Functional Assessments

- Barthel Index (at hospital admission) to characterize functional status and level of dependency [23];
- Clinical Frailty Scale (CFS; metric), assessing frailty on a 9-point ordinal scale (range: 1–9) [24];
- Geriatric Depression Scale (GDS; metric) assessing depressive symptoms and mood on a 15-point scale (range: 0–15) [25] with higher values indicating more depressive symptoms;
- Functional Oral Intake Score (FOIS, metric) assessing patient’s ability to maintain oral intake beyond liquids, including pureed and solid consistencies (total range: 1–7; tube dependent: 1–3, total oral intake with increasing selection of different consistencies: 4–7) [26].

### 2.4. Statistical Analysis

Given the hypothesis-generating nature of the present study, data were interpreted in an exploratory manner. Descriptive statistics (mean, standard deviation (SD), median, and range as appropriate) were used to summarize the data. Distributional assumptions were assessed using the Kolmogorov–Smirnov test; homogeneity of variances was examined using Levene’s test. As the assumption of normality was not met, non-parametric methods (Mann–Whitney U test, Kruskal–Wallis test) were applied for inferential analyses. Nominal and categorical variables are reported as absolute numbers and percentages and were examined for associations using contingency tables and Pearson’s chi-square tests. Bivariate correlation analyses were performed to evaluate potential relationships between two continuous variables.

To facilitate comparison of the study cohort with contemporary reference studies, age groups were defined for the analysis of dental status (DMFT; number of missing and remaining teeth) in accordance with the German Oral Health Study (DMS V and VI): younger older adults (65–74 years) and older adults aged ≥75 years (75–100 years) [9,27].

To identify factors associated with patients’ expressed wish for dental treatment after mutual adjustment, an a priori defined exploratory binary logistic regression model was fitted based on 214 complete cases. The model included indicators of general health status (Barthel Index), medically urgent treatment indications (surgical treatment need), functionally relevant treatment needs (overall prosthodontic treatment need), and pre-admission living situation as a proxy for potential post-discharge support. Variable selection was guided by previous literature and clinical relevance [14,28]. Multicollinearity among independent variables was assessed using the variance inflation factor (VIF). Model performance was evaluated using Cox and Snell’s R^2^ and Nagelkerke’s R^2^, interpreted descriptively, acknowledging that pseudo-R^2^ values in logistic regression are typically lower than R^2^ values in linear models (<0.2: poor fit; ≥0.2: acceptable fit; ≥0.4: good fit) [29]. Model calibration was assessed using the Hosmer–Lemeshow goodness-of-fit test.

To quantify the relative contribution of selected variables as potential predictors of OHRQoL, an a priori defined exploratory multiple linear regression analysis was performed based on 208 complete cases. The selection of independent variables was guided by previous literature identifying relevant determinants of GOHAI in older adults, including depressive symptoms (GDS), dental status (DMFT), prosthodontic treatment need, functional oral intake (FOIS), and living situation [28,30,31,32].

Although GOHAI scores were not normally distributed, linear regression was applied due to its robustness in moderately sized samples and its interpretability, in line with previous studies [32]. Model diagnostics were performed to assess the validity of underlying assumptions, including evaluation of residual distribution (Q–Q plots), influential observations, and homoscedasticity of the error terms. As heteroskedasticity was detected (White’s test), heteroskedasticity-robust standard errors (HC3) were applied to all coefficient estimates. Model fit was evaluated using the coefficient of determination (R^2^), interpreted as small (R^2^ = 0.02), moderate (R^2^ = 0.13), and large (R^2^ = 0.26) explained variance. Effect size was calculated as Cohen’s f^2^, derived from the adjusted R^2^ of the regression model, and interpreted as small (f^2^ = 0.02), medium (f^2^ = 0.15), and large (f^2^ = 0.35) [33].

In addition, a quantile regression analysis (q = 0.5; median GOHAI) was performed using the same set of independent variables to account for the bounded and potentially skewed distribution of GOHAI scores. Given these distributional characteristics, the quantile regression was considered a complementary analytical approach of equal relevance, providing a robustness check for the findings obtained from the linear regression model.

Given the hypothesis-generating nature of the present study, both *p*-values and 95% confidence intervals were interpreted in an exploratory manner. All analyses were performed using IBM SPSS Statistics (version 31.0; IBM Corp., Ehningen, Germany).

## 3. Results

The study included 214 patients (58.4% female) with a mean age of 82 ± 6 years (range: 68 to 96 years). At hospital admission, the cohort showed considerable functional impairment and frailty, as indicated by the low mean Barthel Index (38 ± 19) and the distribution of CFS scores (Table 1).

### 3.1. Dental Status and Clinically Assessed Dental Treatment Need

On average, patients were missing 18 ± 9 teeth (excluding third molars). Overall, 53 patients (24.8%) were edentulous, while only 5 patients (2.3%) had a complete natural dentition. Dentition status varied by age group when stratified according to the age categories used in the German Oral Health Studies (Table 2).

No clinically assessed dental treatment need was identified in 5.1% of patients (*n* = 11). Among dentate patients (*n* = 161), treatment needs were highly prevalent; however, the majority of findings corresponded to elective or functionally relevant conditions rather than urgent indications. Need for SPT was identified in 52.8% of cases, prosthodontic treatment need in 67.7%, and restorative treatment need in 42.9% (Table 3). In contrast, clinically urgent conditions requiring immediate intervention, such as tooth extractions, were present in 26.7% of dentate patients.

Among edentulous patients (*n* = 53), 45.3% required adjustment of existing complete dentures (e.g., relining and/or repair), whereas 22.6% were recommended for the fabrication of new dentures. These needs were considered functionally relevant but not urgent.

A detailed characterization of existing prosthetic restorations and related treatment needs is beyond the scope of the present manuscript and will be reported separately, with a focus on prosthodontic aspects such as denture type and its influence on OHRQoL.

### 3.2. Patients’ Subjective Assessment

In response to the question “Do you currently wish dental treatment?”, 37.9% of patients expressed a wish for dental treatment. Oral health–related quality of life, assessed using the GOHAI, yielded a mean score of 51 ± 7 points (range: 28–60; median 52).

### 3.3. Factors Associated with Patients’ Subjective Assessment of Oral Health

No significant association was found between patients’ self-reported current wish for dental treatment and the presence of clinically assessed dental treatment need (χ^2^(1, *n* = 214) = 1.372, *p* = 0.241, V = 0.08; Chi-square test).

Patients who expressed a wish for dental treatment reported slightly lower GOHAI scores (median = 49) than those who did not (median = 51); however, this difference did not reach statistical significance (U = 4601, z = −1.79, *p* = 0.073, r = 0.12; Mann–Whitney U test).

All variables were examined in univariable analyses to explore potential associations with patients’ expressed wish for dental treatment (see Table 4). Given the number of univariable tests performed, no formal adjustment for multiple comparisons was applied. Therefore, the results should be interpreted as exploratory and with caution regarding potential type I error.

**Figure 1:**
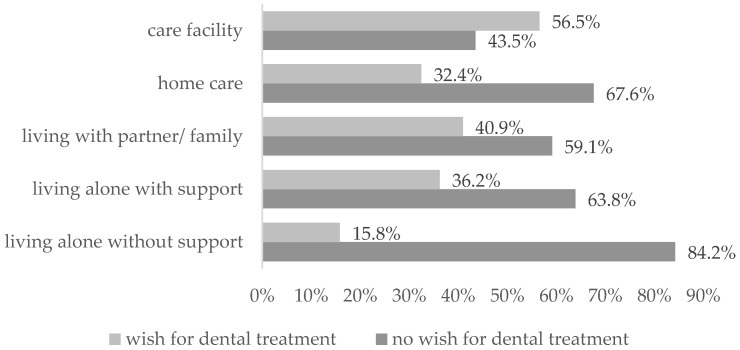
Living situation prior to hospital admission.

The presence of a clinically urgent indication for tooth extraction was significantly associated with patients’ expressed wish for dental treatment (χ^2^(1, *n* = 214) = 4.054, *p* = 0.044, V = 0.138; Chi-square test). No statistically significant associations were observed for the other variables. In particular, pre-admission living arrangement was not significantly associated with treatment desire, although descriptive differences in the distribution of responses were observed. Patients with higher levels of support tended to more frequently express a wish for dental treatment, as shown in Figure 1.

An exploratory binary logistic regression model including 214 cases was conducted to identify potential predictors of patients’ expressed wish for dental treatment.

The model was statistically significant (χ^2^(7) = 18.91, *p* = 0.008; see Table 5). Variance inflation factors ranged from 1.011 (extraction indicated) to 1.018 (Barthel Index), indicating no relevant multicollinearity. However, the number of events relative to the included predictors was limited, which may have affected model stability, particularly for categorical variables with multiple levels such as living arrangement. This is reflected in the width of the confidence intervals presented in Table 5.

Overall model fit was limited, with low explained variance (Cox and Snell’s R^2^ = 0.085; Nagelkerke’s R^2^ = 0.116), indicating modest explanatory power. Calibration, as assessed by the Hosmer–Lemeshow test (χ^2^(8) = 11.39, *p* = 0.18), should be interpreted with caution given the sample size and the limited informative value of this test in such settings.

Taken together, these results should be interpreted as exploratory and hypothesis-generating rather than predictive.

To explore potential determinants of OHRQoL, candidate predictors were examined in univariable analyses with respect to their association with GOHAI (see Table 6).

Pre-admission living arrangement showed a group-dependent association with GOHAI that did not reach statistical significance (H(4) = 9.283, *p* = 0.054, η^2^ = 0.03; Kruskal–Wallis test). Participants living alone without support reported higher GOHAI scores (median 54) compared with those receiving outpatient or home care (median 48; *p* = 0.017, r = 0.32) and those residing in long-term care facilities (median 50; *p* = 0.025, r = 0.35).

Higher FOIS levels were associated with higher GOHAI scores (r = 0.285, *p* < 0.001; Pearson correlation). In addition, patients without prosthodontic treatment need had higher GOHAI scores (median 54) than those with identified prosthodontic treatment need (median 51; U = 3205, Z = −3.58, *p* < 0.001, r = 0.25; Mann–Whitney U test).

To examine the joint effects of multiple variables on OHRQoL, an exploratory multiple linear regression model was fitted based on 208 complete cases (Table 7). The overall model was statistically significant (F(8, 199) = 6.5 4, *p* < 0.001) and demonstrated moderate explanatory power (R^2^ = 0.21; adjusted R^2^ = 0.176; Cohen’s f^2^ = 0.26). However, given the bounded and non-normally distributed nature of GOHAI scores, results should be interpreted with caution. Higher FOIS was significantly associated with higher GOHAI scores (β = 2.15, 95% CI: 1.09 to 3.20; *p* < 0.001). In contrast, the presence of prosthodontic treatment need was associated with lower GOHAI scores (β = −4.13, 95% CI: −6.28 to −1.98; *p* < 0.001), exceeding the minimal important difference for the GOHAI. Patients residing in institutional long-term care facilities had lower GOHAI scores compared to those living alone without support (β = −4.32, 95% CI: −8.49 to −0.16; *p* = 0.042), whereas other living arrangements were not significantly associated with GOHAI. No statistically significant associations were observed for GDS or DMFT. Variance inflation factors ranged from 1.05 to 3.65, indicating no relevant multicollinearity.

To complement the linear regression analysis and to account for the bounded and potentially skewed distribution of GOHAI scores, a quantile regression model (q = 0.5) was performed using the same set of predictors (Table 8). The direction and magnitude of key associations were broadly consistent across both models. In the quantile regression, prosthodontic treatment need was associated with a reduction in median GOHAI (β = −2.95, *p* = 0.041), while higher FOIS remained positively associated (β = 2.18, *p* = 0.002). Living in outpatient care was associated with lower median GOHAI (β = −6.09, *p* = 0.019), whereas other categories did not reach statistical significance.

Taken together, both modeling approaches yielded comparable patterns, supporting the robustness of the main findings, although the results should be interpreted as exploratory.

## 4. Discussion

This study provides three main findings. First, clinically assessed dental treatment need was highly prevalent among older adults undergoing acute geriatric complex treatment. Second, only a minority of patients expressed a wish for dental treatment, and no significant association was observed between clinically assessed need and patient-reported wish. Third, selected factors—particularly prosthodontic treatment need, functional oral intake (FOIS), and living situation—were associated with OHRQoL, although the overall explanatory power of the models was limited.

Compared with population-based reference data from the German Oral Health Studies (DMS V and VI), the present cohort showed less favorable dental status [9,27]. However, these comparisons should be interpreted cautiously, as the study population represents a highly selected group of hospitalized, multimorbid older adults. In addition, comparisons were based on different reference studies for separate age groups due to data availability, which further limits direct comparability. Accordingly, these data should be considered contextual rather than epidemiological.

Although nearly all patients exhibited some form of dental treatment need, only a subset had conditions likely to be medically urgent during hospitalization, such as extraction indications [34]. The majority of findings were functionally relevant or elective in nature [35]. This distinction is essential for interpreting the observed discrepancy between clinically assessed treatment need and patient-reported wish for dental treatment, as patients may reasonably deprioritize non-urgent or asymptomatic conditions during acute illness.

Only approximately one third of patients reported a wish for dental treatment. While patients expressing such a wish tended to report lower GOHAI scores, this difference did not reach statistical significance and did not exceed the minimal important difference (MID) [22]. The overall mean GOHAI in the present cohort was 51 ± 7 points, which is below reference values reported in the literature (approximately 54 points), typically derived from healthier community-dwelling older adults [36]. Accordingly, no clinically meaningful difference in OHRQoL between these groups can be inferred. This finding should not be interpreted as the absence of oral health burden but rather as a reflection of the clinical context in which priorities are shaped, particularly in the presence of competing health concerns during acute hospitalization.

Patients undergoing acute geriatric complex treatment are typically characterized by advanced age, multimorbidity, frailty, and reduced functional status. In this context, competing medical priorities, limited physical and cognitive resources, and reduced perceived short-term benefit may influence the prioritization of dental care. As previously described, frail older adults may consciously allocate limited resources to more pressing health concerns rather than oral healthcare [37].

From a conceptual perspective, the discrepancy between clinically assessed treatment need and patient-reported wish may be understood in light of Andersen’s behavioral model of health service utilization, which emphasizes that healthcare use is shaped not only by need but also by predisposing characteristics and enabling resources [38]. In the setting of acute hospitalization, these interacting factors—including health status, dependency, and availability of support—may substantially influence patients’ expressed treatment preferences.

Exploratory analyses suggested that medically urgent conditions, such as extraction need, were more closely associated with patients’ expressed wish for treatment than non-urgent findings. This supports the assumption that perceived urgency plays an important role in shaping patient priorities. Similarly, living situation appeared to influence treatment wish, with patients embedded in supportive environments more likely to express interest in dental care. This observation is consistent with previous findings that access to dental services is influenced by enabling factors such as social support, transportation, and caregiving structures [39,40,41]. However, these associations should be interpreted cautiously, as multivariable models showed limited explanatory power and were not designed for prediction.

With regard to OHRQoL, prosthodontic treatment need and functional oral intake (FOIS) emerged as potential key associated factors. Patients with prosthodontic treatment need showed lower GOHAI scores, exceeding the minimal important difference in the linear model, whereas the corresponding estimate in the quantile regression was smaller and only marginally reached this threshold. This suggests a potentially clinically relevant, but not uniformly consistent, association.

The relationship between prosthodontic treatment need and OHRQoL may be interpreted in the context of functional oral impairment. In older adults, inadequate or poorly fitting dental prostheses can compromise masticatory efficiency and comfort, potentially leading to adaptations in dietary behavior, including a shift toward softer or less diverse food consistencies [42,43,44,45]. This may, in turn, be reflected in reduced functional oral intake as captured by the FOIS. In the present study, higher FOIS scores were consistently associated with better OHRQoL, highlighting the role of oral intake as a functional link between oral health and perceived well-being. These findings are in line with previous research demonstrating associations between oral function, swallowing ability, and quality of life in older adults [42,46,47].

More broadly, oral health, nutritional status, and general health are closely interconnected in older adults [10,11,12,13,48]. Impaired oral function and reduced dietary diversity have been associated with an increased risk of malnutrition and frailty [46,49,50]. Conversely, increasing frailty and dependency may negatively affect oral hygiene, prosthesis maintenance, and access to dental care, suggesting a bidirectional relationship between oral health and general health status.

Patients requiring higher levels of care or living in institutional settings reported lower OHRQoL, which may reflect broader aspects of health status and dependency rather than dental factors alone. This observation is consistent with previous studies showing that increasing care dependency and institutionalization are associated with reduced autonomy, greater health burden, and lower quality of life in older adults [8,12,37]. However, these associations should be interpreted as associative rather than causal.

From a clinical perspective, the findings suggest that dental screening in acute geriatric settings may help identify previously unrecognized oral conditions, particularly those that are medically relevant or functionally impairing. However, the present study does not provide evidence regarding the feasibility, effectiveness, or patient benefit of implementing dental interventions during hospitalization. Any implications regarding the integration of dental care into acute geriatric treatment should therefore be considered hypothesis-generating.

Overall, the findings underscore the importance of distinguishing between different types of dental treatment need and between clinically assessed need and patient-reported preferences. The observed discrepancy should not be interpreted as a lack of patient awareness but rather as a reflection of contextual, functional, and methodological factors.

This study has several limitations that should be considered when interpreting the findings. First, potential selection bias may have influenced the study sample. Although all patients undergoing acute geriatric complex treatment during the study period were screened, inclusion required the ability to provide informed consent and complete self-reported assessments. Consequently, patients with severe cognitive impairment, acute delirium, or high clinical burden were underrepresented. In addition, refusal to participate, fatigue, and early discharge may have further affected participation. As a result, the study cohort likely represents a relatively more stable subgroup of hospitalized older adults, which limits generalizability to more vulnerable populations. Furthermore, the single-center design may additionally restrict the transferability of the findings to other clinical settings.

Second, multivariable analyses were based on complete-case datasets without formal assessment of missing data patterns. As missingness in frail inpatient populations is unlikely to be random, this approach may have introduced bias and affected the representativeness of the analytic sample, particularly in analyses combining patient-reported and clinical variables.

Third, patient-reported wish for dental treatment was assessed using a single dichotomous item, which provides only limited insight into patient preferences. This measure does not capture ambivalence, conditional willingness, or structural barriers to care, and may therefore have led to misclassification, particularly under conditions of acute illness.

Fourth, the concept of “dental treatment need” encompasses a heterogeneous range of conditions, including urgent, functionally relevant, and elective interventions. The aggregation of these distinct categories into a single construct may have contributed to an overestimation of overall treatment need and may have influenced the observed discrepancy between clinically assessed need and patient-reported wish.

Fifth, dental examinations were conducted under bedside conditions without the use of a dental chair or radiographic imaging, which may have limited diagnostic accuracy, particularly for subclinical or radiographically detectable findings. In addition, no formal inter- or intra-examiner reliability statistics (e.g., kappa or intraclass correlation coefficients) were calculated. Although examiners were calibrated through joint assessments and consensus discussions, the absence of quantitative agreement measures may have introduced variability in outcome classification, particularly for examiner-dependent endpoints.

Finally, the statistical modeling approach has several limitations. Although covariates were selected based on clinical relevance and prior evidence, analyses were exploratory and not designed for confirmatory inference. The number of complete cases available for regression analyses was reduced due to missing data, which may have further affected representativeness. Moreover, GOHAI scores are bounded and deviated from normality, which may limit the assumptions underlying linear regression. While robust standard errors and complementary quantile regression analyses were applied, these approaches do not fully address all distributional constraints. The observed effect sizes and explained variance indicate only moderate model performance, and results should therefore be interpreted with caution. Although no critical multicollinearity was detected, the higher VIF observed for the living situation variable suggests some overlap between predictors, which may have affected the precision of individual estimates. In addition, the cross-sectional design precludes any conclusions regarding causality or the directionality of the observed associations.

## 5. Conclusions

Clinically assessed dental findings were common in this selected cohort of older adults undergoing acute geriatric complex treatment, whereas a single-item measure of treatment desire captured only part of patients’ oral health concerns during hospitalization. Targeted dental screening focusing on urgent and functionally relevant conditions may therefore deserve further evaluation in acute geriatric settings.

## Figures and Tables

**Table 1:** Study cohorts’ characteristics (*n* = 214).

Variable	Category					
General health characteristics						
Sex (*n*/%)	male: 89 (41.6); female: 125 (58.4)					
Age (years, mean ± SD)	82 ± 6 years (median: 83, range: 68–96 years)					
No. of medications daily (mean ± SD)	13 ± 4 (median: 13, range: 4–23)					
Level of care prior to admission (*n*/%)	None	Level 1	Level 2	Level 3	Level 4	Level 5
	71 (33.2)	17 (7.9)	70 (32.7)	49 (22.9)	4 (1.9)	3 (1.4)
Living situation prior to admission (*n*/%)	Living alone without support	Living alone with support (family, friends)	Living with partner/family	Outpatient/home care	Care facility (institutional long-term care)	
	19 (8.9)	47 (22.0)	88 (41.1)	37 (17.3)	23 (10.7)	
Geriatric and functional assessments						
Barthel Index (admission, mean ± SD)	38 ± 19 (median: 40, range 0–90)					
Geriatric Depression Scale (GDS, *n*/%)	Normal range (0–5 points)		Mild to moderate depressive symptoms (6–10 points)		Severe depressive symptoms	Missing values
					(11–15 points)	
	134 (62.6)		72 (33.6)		5 (2.3)	3 (1.4)
Clinical Frailty Scale (CFS, *n*/%)	None	Grade 3	Grade 4	Grade 5	Grade 6	Grade 7
	13 (6.1)	2 (0.9)	7 (3.3)	125 (58.4)	52 (24.3)	15 (7.0)
Functional Oral Intake Score (FOIS, *n*/%)	Level 2	Level 4	Level 5	Level 6	Level 7	Missing values
	1 (0.5)	7 (3.3)	36 (14.9)	79 (36.9)	88 (41.1)	3 (1.4)

*n* = number, SD = standard deviation.

**Table 2:** Dentition status by age group, based on the age stratification used in the German Oral Health Study (DMS V).

Variable		Age Group	
		65–74 Years * (*n* = 30)	75–100 Years * (*n* = 184)
DMFT (mean ± SD)		24 (±7)	24 (±5)
Missing teeth (mean ± SD)		16 (±11)	18 (±9)
Dentition status (*n*/%)	Edentulous	10 (33.3)	43 (23.4)
	Partially dentate	19 (63.3)	137 (74.5)
	Fully dentate	1 (3.3)	4 (2.2)

DMFT: decayed, missing, filled teeth; *n*: number. * Age-specific comparisons were based on DMS V and DMS VI data; however, differences in available age strata required a mixed-reference approach, which should be considered when interpreting the results.

**Table 3:** Type of clinically assessed dental treatment need, categorized as restorative, periodontal, surgical, and prosthodontic treatment in dentate patients (*n* = 161).

Clinical Urgency Assessment	Treatment Required	Specification (*n* [%])					
Elective	Restorative treatment need	No need		Restorative therapy			
		92 (57.1)		69 (42.9)			
	Periodontal treatment need	No need		PTC		SPT	
		11 (6.8)		65 (40.4)		85 (52.8)	
Functional	Prosthetic treatment need	No need	Repair/relining	New prosthesis required			Not assessable
				FPD	RPD	FPD + RPD	
		52 (32.3)	51 (31.7)	11 (6.8)	27 (16.8)	14 (8.7)	6 (3.7)
Urgent	Surgical treatment need	No need		Tooth extraction requirement			
		118 (73.3)		43 (26.7)			

*n* = number, PTC = professional tooth cleaning, SPT = systematic periodontal therapy, FPD = fixed partial denture, RPD = removable partial denture.

**Table 4:** Summary of test results for potential factors associated with patients’ expressed wish for dental treatment.

Variable	*p*-Value	Variable	*p*-Value
General health status			
Age	0.397 *	Living situation	0.084 °
Sex	0.630 °	Level of care (yes/no)	0.345 °
Geriatric and functional assessments			
Barthel Index	0.103 *	CFS	0.688 °
GDS	0.981 °	FOIS	0.659 °
Dentition status and dental treatment demand			
DMFT	0.232 *	Dentition status	0.495 °
Restorative treatment need	0.797 °	Prosthodontic treatment need	0.185 °
Periodontal treatment need	0.735 °	Surgical treatment need	0.044 °

* Mann–Whitney U test, ° Pearson’s chi-square test. GDS: Geriatric Depression Scale, CFS: Clinical Frailty Scale. FOIS: Functional Oral Intake Score, DMFT: decayed, missing, filled teeth.

**Table 5:** Binary logistic regression assessing the predictive value of the Barthel Index, prosthodontic treatment need, extraction indication, and pre-admission living arrangement for patients’ expressed desire for dental treatment.

Variable		Odds Ratio	95% CI		*p*-Value
Barthel Index (admission)		1.02	1.00	1.04	0.030
Overall prosthodontic treatment need *		1.01	0.53	1.93	0.973
Extraction indicated **		2.32	1.13	4.78	0.022
Living situation ***	Living alone with support (family/friends)	3.86	0.95	15.75	0.060
	Living with partner/family	4.42	1.16	16.78	0.029
	Outpatient/home care	3.24	0.77	13.76	0.110
	Care facility (institutional long-term care)	11.38	2.39	54.30	0.002

* Reference category: no prosthodontic treatment need. ** Reference category: no surgical treatment need (no extraction indicated). *** Reference category: living alone without support.

**Table 6:** Summary of test results for potential factors associated with the GOHAI score.

Variable	*p*-Value	Variable	*p*-Value
General health status			
Age	0.971 *	Living situation	0.054 **
Sex	0.219 °	Level of care (yes/no)	0.074 °
Geriatric and functional assessments			
Barthel Index (admission)	0.212 *	CFS	0.224 **
GDS	0.073 **	FOIS	<0.001 *
Dentition status and dental treatment demand			
DMFT	0.001 *	Dentition status	0.704 **
Restorative treatment need	0.885 °	Prosthodontic treatment need	<0.001 **
Periodontal treatment need	0.525 **	Surgical treatment need	0.447 °

* Bivariate correlation ° Mann–Whitney U test, ** Kruskal–Wallis test. GDS: Geriatric Depression Scale, CFS: Clinical Frailty Scale. FOIS: Functional Oral Intake Score, DMFT: decayed, missing, filled teeth.

**Table 7:** Results of the multiple linear regression analysis using heteroskedasticity-robust standard errors (HC3 method) to identify potential predictors of GOHAI.

Variable		Regression Coefficient	95% CI		*p*-Value
GDS		−0.15	−0.46	0.16	0.327
FOIS		2.15	1.09	3.20	0.000
Living situation *	Living alone with support (family/friends)	−1.23	−4.96	2.51	0.517
	Living with partner/family	−0.94	−4.39	2.51	0.592
	Outpatient/home care	−3.10	−6.97	0.76	0.115
	Care facility (institutional long-term care)	−4.32	−8.49	−0.16	0.042
DMFT		−0.03	−0.21	0.15	0.773
Prosthodontic treatment need **		−4.13	−6.28	−1.98	0.000

* Reference category: living alone without support. ** Reference category: no prosthodontic treatment need.

**Table 8:** Quantile regression (q = 0.5) as sensitivity analysis to identify potential predictors of the median GOHAI score.

Variable		Regression Coefficient	95% CI		*p*-Value
GDS		−0.25	−0.65	0.16	0.228
FOIS		2.18	0.79	3.57	0.002
Living situation *	Living alone with support (family/friends)	−1.58	−6.49	3.34	0.527
	Living with partner/family	−1.16	−5.70	3.39	0.617
	Outpatient/home care	−6.09	−11.19	−1.04	0.019
	Care facility (institutional long-term care)	−4.48	−9.96	1.00	0.109
DMFT		0.04	−0.20	0.27	0.756
Prosthodontic treatment need **		−2.95	−5.78	−0.12	0.041

* Reference category: living alone without support. ** Reference category: no prosthodontic treatment need.

## Data Availability

Data used and/or analyzed during the current study are available from the corresponding author on reasonable request.
